# *Bacillus* predominates in the *Ophiocordyceps pseudolloydii*-infected ants, and it potentially improves protection and utilization of the host cadavers

**DOI:** 10.1007/s00203-022-03385-9

**Published:** 2023-01-05

**Authors:** Hao-Yu Kuo, Ming-Chung Chiu, Jui-Yu Chou

**Affiliations:** Department of Biology, National Changhua University of Education, 500, Changhua, Taiwan

**Keywords:** Bacillus, *Ophiocordyceps pseudolloydii*, Pathogenic fungi, Bacterial community, Nematode

## Abstract

The bacterial communities that colonize the cadaver environment of insects infected and killed by parasitic fungi can be selected by the sympatric fungi and provide novel impacts. In this study, we found that *Bacillus cereus/thuringiensis* predominate the bacterial community in *Dolichoderus thoracicus* ant cadavers colonized by *O. pseudolloydii*. The most predominant bacterial strains in these ant cadavers were hemolytic and able to produce hydrolytic enzymes for digesting the ant tissue. A relatively intense lethal effect on the co-cultured nematode was displayed by a hemolytic strain. Moreover, the antagonistic effect against pathogenic fungi detected in the bacteria sympatric with *O. pseudolloydii* was reported here. Naphthoquinones have been shown to confer antibacterial activities and produced by the ant-pathogenic *Ophiocordyceps* fungi. However, our results did not show the naphthoquinone tolerance we expected to be detected in the bacteria from the ant infected by *O. pseudolloydii*. The bacterial diversity in the samples associated with *O. pseudolloydii* infected ants as revealed in this study will be a step forward to the understanding of the roles playing by the microbial community in the native habitats of *O. pseudolloydii*.

**Supplementary Information:**

The online version contains supplementary material available at 10.1007/s00203-022-03385-9.

## Introduction

Among the sympatric organisms, parasites and microbes in an individual host form a unique relationship. In addition to the simple competition, the interaction between sympatric microbes and parasites usually depends on their functions and biological properties, including nutritional role, immunomodulation property, and contribution to the pathogenesis (Leung et al. 2018). Certain facultative microbial species can also directly colonize host bodies independent of an intermediary parasite (Drew et al. 2021). In recent years, the development of high-throughput sequencing has promoted the description of microbial community composition. Although biological properties and ecological functions of the microbes can be estimated by the phylogeny, the commonly used marker, known as the V3/V4 region in the 16S rDNA gene, is sometimes insufficient to distinguish the closely related taxa (Wei et al. 2019). In addition, functional divergence can also be found in closely related taxa. In this study, *Bacillus cereus/thuringiensis* was found to predominate the bacterial community in ant cadavers colonized by *O. pseudolloydii*, we aim to investigate their biological properties in the context of their adaptation to the microenvironment.

In the recent literature, increasing attention is being paid to the potential for host- and parasite-associated microbiota to influence and shape host − parasite interactions. It has been suggested that the nature of parasite − microbe interactions may have a critical effect on the parasite's fitness and further effects on the pathogenesis (Dheilly et al. 2019). An RNA virus of the braconid wasp parasite, *Dinocampus coccinellae*, is stored in the oviduct of parasitoid females, replicates in parasitoid larvae, and is transmitted to its coccinellid host, *Coleomegilla maculate*, during the host behavioral manipulation (Dheilly et al. 2015). However, the changes in ladybeetle behavior are most likely caused by virus replication in the cerebral ganglia instead of direct manipulation by the parasitic wasp (Dheilly et al. 2015). Entomopathogenic bacteria from the genera *Photorhabdus* and *Xenorhabdus* are closely related Gram-negative bacteria and establish obligate mutualistic associations with soil nematodes of *Steinernema* and *Heterorhabditis* to facilitate insect pathogenesis (Ferreira and Malan 2014). These symbiotic bacteria are able to produce toxins to cause insect death and enzymes to overcome host immunity, degrade host tissues, and make the nutrient-rich insect cadavers available for the developing nematodes (Lu et al. 2016). Similarly, an air-borne plant-pathogenic fungus, *Fusarium graminearum*, has a cooperative interaction with a seed-borne plant-pathogenic bacterium, *Burkholderia glumae*. It even results in the promotion of bacterial survival, bacterial and fungal dispersal, and disease progression on rice plants (Jung et al. 2018). These beneficial effects of bacterial − fungal interactions have also been described for *B. rhizoxinica* and its fungal host and cause rice seedling blight (Partida-Martinez and Hertweck 2005; Partida-Martinez et al. 2007).

In middle Taiwan, two parasitoid fungi, *O*. *pseudolloydii* and *O. unilateralis s. l.*, infect ants in the same broad-leaved forest (Chung et al. 2017). The infected ants displayed a similar parasitic manipulation, climbing upward toward the canopy and dying on the tree leaves. In our previous work, *O. unilateralis s. l.* broadly infected eight sympatric ant species, with a preference for a principal host, *Polyrhachis moesta*, as shown by the relatively higher infection rate, success in the behavioral manipulation, and growth rate (Chung et al. 2017). Examination of the bacterial community in the principal host, as well as an alternative host, *P. wolfi*, and in both cases, *B. cereus/thuringiensis* was the predominant species (Tu et al. 2021). In contrast, the host of *O. pseudolloydii* is more specific than that of *O. unilateralis s. l.* because only one ant species, *D*. *thoracicus*, was found to be infected on the tree leaves (Chung et al. 2017). The infected *D*. *thoracicus* ants were covered in a dense matrix of fungal hyphae, securing the abdominal segments and the mandibles to the underside of plant leaves. After several weeks, the fungal fruiting bodies grow from the ant's head and rupture, releasing fungal spores onto the forest floor below. This process is theoretically at high energy costs, but the results of histological cross sections indicated that the fungus does not actually invade the leaf tissue; the fungus only attaches to the plants (Chung et al. 2017). It means the energy for fungal growth is highly dependent on and mostly generated from the infected ant cadavers. Considering the similar parasitic biology between *O*. *pseudolloydii* and *O. unilateralis s. l.*, we expect to see a similar pattern of component species of sympatric bacteria in *O*. *pseudolloydii*-infected ant cadavers to *O. unilateralis s. l.-*infected ant cadavers. The difference in host species belonging to different subfamilies (*D*. *thoracicus*: Dolichoderinae; *Polyrhachis*: Formicinae) might also influence the biological properties of the sympatric bacteria. In this study, we characterized the bacterial community by a culture-dependent method and assessed the hemolysis reaction, resistance to naphthoquinones, production of hydrolytic enzymes, and antagonistic effect against pathogenic fungi.

## Materials and methods

### Sample collection

**Fig. 1:**
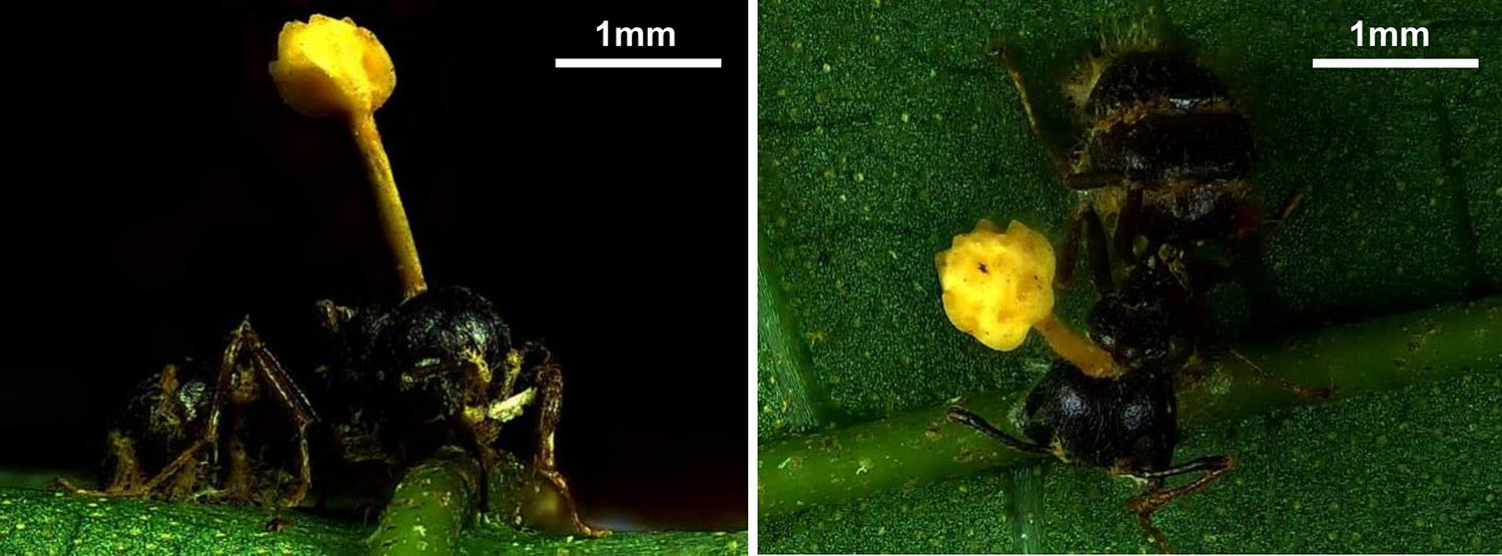
*Ophiocordyceps pseudolloydii* infected *Dolichoderus thoracicus* with the stroma growing from the ant cadaver. The specimens were collected from the Lianhuachi Research Center, Taiwan, and photographed in the laboratory. Left: side view; Right: top view

Samples were collected from the Lienhuachi Experimental Forest (Permission no.: 1082272516), an evergreen broadleaf forest in central Taiwan (23°55′7″N 120°52′58″E) in 2020. Ant cadavers infected by *O*. *pseudolloydii* were carefully removed by cutting the leaf and placing it into a 50-mL conical centrifuge tube, which was then transported to the laboratory. Only cadavers in which the fungal growth stage preceded the development of pale yellow perithecia, which theoretically has the highest biological activity, were collected (Fig. 1). Altogether, 24 infected *D*. *thoracicus* samples were collected and examined in this study.

### Bacterial isolation and cultivation

The protocols used for isolating bacteria were described in our previous work (Tu et al. 2021). In brief, sample surfaces were cleaned by agitation in 600 μL of sterilized water using a vortex mixer (AL-VTX3000L, 114 CAE Technology Co., Ltd., Québec, Canada), soaked with 600 μL of 70% ethanol, washed twice with 600 μL of sterilized water, and then vortexed in 400 μL of sterilized water. Two hundred microliters of the supernatant was spread homogeneously onto a Luria − Bertani (LB) agar plate (25 g LB broth and 15 g agar per liter) to confirm the absence of live bacteria. The cleaned samples were homogenized in 200 μL of water to release the internal bacteria and cultured on LB agar plates at 28 °C for 2 days. Approximately equal numbers of the bacterial isolates from each of the ant individuals were picked randomly with sterile toothpicks and suspended individually in the LB medium supplemented with 15% (v/v) glycerol and maintained at – 80 °C until examination. In total, 137 bacterial isolates were collected (~ 5 to 6 isolates per ant sample). In addition to the bacterial isolates from the ant bodies, 60 bacterial isolates from soil, leaves, and air in the same locality were sampled to examine the tolerance to naphthoquinones (see below) using the procedure mentioned above but without initial cleaning and sterilizing of the sample surface. Briefly, a sterilized steel corer was inserted into soil, from which soil samples were taken from ~ 5 cm depth and then placed into sterile polycarbonate centrifuge tubes. For leaf sampling, leaves from different size and without visible disease symptoms or physical damage were collected. Leaves were randomly taken to obtain a representative composed sample. For air sampling, lid opened plates with LB agar medium were placed on the ground for 5 min. When the expected sampling time was finished, the sample plates were covered by lids and transported to the laboratory for culture.

### Bacterial identification

Genomic DNA was extracted from the bacterial isolates cultured in LB medium at 28 °C overnight. Randomly amplified polymorphic DNA (RAPD) analysis with the primer 5′-GAGGGTGGCGGTTCT-3′ (Huey and Hall 1989) was used to determine the bacterial strain. The PCR condition followed our previous work: initial denaturation at 95 °C for 5 min, 40 cycles of amplification, including denaturation at 95 °C for 1 min, annealing at 42 °C for 30 s, and extension at 72 °C for 1 min, followed by a final extension at 72 °C for 10 min. The RAPD pattern was checked by gel electrophoresis of the PCR products on a 2% agarose gel. Bacterial isolates with the same RAPD pattern were determined as the same strain and coded with "JYCB," followed by a series of numbers (e.g., JYCB1491).

The taxonomic status of each strain was determined by sequencing the V3/V4 hypervariable regions of the 16S rDNA gene. The sequence was first amplified by PCR with the primer set (8F: 5′-AGAGTTTGATCCTGGCTCAG-3′ and 1541R: 5′-AAGGAGGTGATCCAGCCGCA-3′) under the following condition: initial denaturation at 95 °C for 5 min, 40 cycles of amplification, including denaturation at 95 °C for 1 min, annealing at 55 °C for 30 s, and extension at 72 °C for 1 min 45 s, followed by a final extension at 72 °C for 10 min (Edwards et al. 1989; Turner et al. 1999), and sequenced at Genomics, Inc. (New Taipei City, Taiwan).

To identify the taxon, the bacterial strains were first clustered into the same clades according to the sequence dissimilarity (< 0.01) calculated by the unweighted pair group method with arithmetic mean (UPGMA) using MEGA X (Kumar et al. 2018). Each clade was considered as an operational taxonomic unit in this study. The species was judged by the basic local alignment search tool (BLAST) method against nucleotide sequences in the National Center for Biotechnology Information (NCBI) nucleotide database (https://ftp.ncbi.nlm.nih.gov/blast/db/), updated May 17, 2021.

Each strain was first labeled with the species of the sequence with the highest BLAST identity, which was ranked by expect value (E-value), bit score, percentage of identical matches, and alignment length (https://www.ncbi.nlm.nih.gov/BLAST/tutorial/Altschul-1.html). If multiple sequences from the database were found to be the same in the identity indexes, the bacterial strain was labeled by the species that appeared most frequently. In the cases of the clades containing several bacterial strains, the species were judged by the bacterial species found most frequently in the strains belonging to the clade.

The 60 bacterial isolates collected from the environment were examined using the RAPD method and a *Bacillus*-specific primer set (5’-CTTGCTCCTCTGAAGTTAGCGGCG-3’ and 5’-TGTTCTTCCCTAATAACAGAGTTTTACGACCCG-3’), with the PCR conditions suggested by Nakano et al*.* (Nakano et al. 2004). Twenty of the bacterial isolates (10 *Bacillus* and 10 non-*Bacillus*) with different RAPD patterns were collected for their resistance to naphthoquinones (see below).

### Biological properties of bacterial isolates from infected ants

#### Selected strains

All the bacterial strains isolated from ant cadavers were selected for examining the hemolysis reaction. Among multiple isolates belonging to a strain, one isolate was selected randomly for the examination. To characterize the biological properties, including hydrolytic activity, repellence against pathogenic fungi, and resistance to naphthoquinone derivatives, 20 bacterial strains (Fig. S1) were selected according to the UPGMA analysis of the sequences, which maximally cover the diverging clades. The 20 bacterial strains included 13 strains of the predominant species (*B. cereus/thuringiensis*, clade 7), and one strain from each of the clades 1, 6, 8, 9, 12, 17, and 18 with relatively lower abundance (Table 1, Fig. S1). We selected one hemolytic strain (JYCB1618) and one non-hemolytic strain (JYCB1543) to examine the effect of co-cultured nematodes because the antagonistic effect might have the potential to be a repellent against the soil pests. For survival studies, nematodes were observed daily for 5 days.

**Table 1:** The activity of hydrolytic enzymes produced by the bacteria from infected *Dolichoderus thoracicus*

Bacterial strains	Clade	Species	Chitinase activity	Protease activity	Lipase activity	Esterase activity
JYCB1497	7	*Bacillus cereus/thuringiensis*	–	+	–	–
JYCB1502			–	+	+	+
JYCB1510			–	+	–	+
JYCB1527			–	+	+	–
JYCB1532			–	+	–	–
JYCB1538			–	+	+	+
JYCB1543			–	+	+	–
JYCB1547			–	+	+	–
JYCB1552			–	+	+	–
JYCB1587			+	+	+	+
JYCB1611			+	+	–	+
JYCB1618			+	+	+	+
JYCB1624			+	+	–	–
JYCB1492	6	*Bacillus gibsonii*	–	–	+	–
JYCB1504	17	*Bacillus subtilis*	–	+	–	+
JYCB1551	18	*Bacillus* sp.	–	–	+	–
JYCB1568	12	*Lysinibacillus xylanilyticus*	–	–	–	–
JYCB1494	9	*Oceanobacillus sojae*	–	–	–	–
JYCB1560	1	*Paenibacillus* sp.	–	–	+	+
JYCB1493	8	*Priestia megaterium*	–	+	+	+

(+  positive; – negative)

#### Hemolysis reaction

For hemolysis reaction tests, one 3-µL drop of the log-phase bacterial suspension was placed onto tryptic soy agar (TSA) plates (15 g pancreatic digest of casein, 5 g soybean meal, 5 g NaCl, and 15 g agar, with final pH of 7.3), which was mixed with 5% defibrinated sheep blood after it had cooled down to approximately 50 °C. The hemolysis reaction was determined by the formation of clean (β-hemolysis) or greenish (α-hemolysis) hemolytic zones, or no such zone (γ-hemolysis, non-hemolytic) around the bacterial colonies after incubation at 28 °C for 1–2 days (Baumgartner et al. 1998).

### Resistance to naphthoquinones

To examine the resistance of bacterial isolates to naphthoquinones, the growth of 13 bacterial strains from the predominant clade (clade 7) and seven low-abundant clades (clade 1, 6, 8, 9, 12, 17, 18) isolated from the ant hosts, and the 20 environmental bacterial isolates (10 *Bacillus* and 10 non-*Bacillus*) was compared using two naphthoquinones, respectively. As the fungal naphthoquinones are currently not purified and commercialized, the two naphthoquinones prepared for the experiment, plumbagin (de Paiva et al. 2003) and lapachol (Eyong et al. 2006), were those found in plants. The two naphthoquinones prepared for the experiment were dissolved in 30% dimethyl sulfoxide (DMSO) − 70% water solution. The naphthoquinone concentrations to be used for testing the bacterial resistance to this family of secondary metabolites were determined in a pilot test conducted as following; the naphthoquinones underwent serial dilutions (plumbagin: from 90–0.09 µg/mL; lapachol: from 257.5–0.5 µg/mL) from which the most suitable concentrations were picked based on the most obvious difference of growth rate among three randomly selected bacteria from the ant host and three from the environment. Based on these results, the concentrations of 45 µg/mL (plumbagin) and 64.5 µg/mL (lapachol) were used for further tests.

In this experiment, the bacterial isolates were first inoculated in LB medium at 20 °C (the mean annual temperature in Lianhuachi Research Center, where the infected ants were collected) overnight and then refreshed to the exponential phase with LB medium for 3 h. The bacterial concentration was adjusted to ~ 1.5 × 10^8^ cells/mL. Next, 10 μL of the bacterial suspension and 180 μL of Mueller − Hinton broth medium (Sigma-Aldrich) were added to either 10 μL of the naphthoquinone solution or 10 μL of the 30% DMSO − 70% water solution for the control. The growth of bacterial isolates at 20 °C was monitored by measuring the optical density at 600 nm (OD_600_) with a Multiskan GO microplate spectrophotometer (Thermo Scientific) every hour for 12 h. Each combination of bacterial isolate and naphthoquinone or control was replicated twice.

The resistance index of each bacterial isolate was calculated by the following formula: [OD_naphthoquinone_ – OD_DMSO_]/[OD_naphthoquinone_ + OD_DMSO_]. The resistance index was analyzed using a linear mixed model with the resource (*Bacillus* from the ant host, *Bacillus* and non-*Bacillus* from the environment) as the fixed effect, the bacterial isolate and experimental replication as random effects, and growth time (5 − 12 h) as a nest effect. The significance of the fixed effect was examined by the likelihood ratio test with the model removing the fixed-effect term. Post hoc tests were undertaken using Tukey's all-pair comparisons. The model building and hypothesis tests were conducted using the "lme4" and "multcomp" packages in R.

### Production of hydrolytic enzymes

The productions of chitinase, proteinase, lipase, and esterase were examined with four different types of solid media: (1) chitinase detection medium (solid medium with 0.3 g MgSO_4_.7H_2_O, 3 g (NH_4_)_2_SO_4_, 2 g KH_2_PO_4_, 1 g citric acid monohydrate, 0.15 g bromocresol purple, 200 μL Tween 80, 4.5 g colloidal chitin, and 1 L deionized water with 1.5% [w/v] agar, with final pH of 4.7), (2) skim milk agar (solid medium with 2% [w/v] agar, 28 g skim milk powder, 5 g casein enzymic hydrolysate (Tryptone), 2.5 g yeast extract, 1 g dextrose, and 1 L deionized water), (3) lipase agar (solid medium with 2% [w/v] agar, 0.1 g phenol red, 1 g CaCl_2_, 10 mL olive oil, and 1 L deionized water, with final pH of 7.4), and (4) esterase agar (solid medium with 2% [w/v] agar, 0.1 g phenol red, 1 g CaCl_2_, 10 mL tributyrin, and 1 L deionized water, with final pH of 7.4). A 3-µL drop of the exponential-phase bacterial suspension was cultured on each of the media, and the production of the hydrolytic enzymes was determined by purple zones for chitinase activity (Agrawal and Kotasthane 2012; Chi et al. 2009), clearance zones for proteinase activity (Cattelan et al. 1999), and yellow zones for lipase and esterase activity (Ramnath et al. 2017). All the experiments were carried out in the dark at 20 °C with three replicates. The plates were observed on days 3, 5, and 7 of the incubation.

### Antagonism against pathogenic fungi isolated from the insect cadavers

Here, we are not able to perform the experiment to see whether the bacteria against *O*. *pseudolloydii* itself. The main reason is that due to it is not yet possible to culture *O. pseudolloydii* under our laboratory conditions. Thus, growth inhibition was detected in most of the bacteria strains against three pathogenic fungi, including *Aspergillus nomius* (isolated from *D*. *thoracicus*), *Trichoderma asperellum* (isolated from the litchi stink bug, *Tessaratoma papillosa*), and *Purpureocillium lilacinum* (isolated from *T. papillosa*). They were cultured on potato dextrose agar (PDA) plates for 4 (*A. nomius*, *T. asperellum*) or 10 (*P. lilacinum*) days at 28 °C, until the mycelia covered approximately 80% of the plate. A piece of mycelium (approximately 5 × 5 mm^2^) was seeded in the center of a TSA plate and surrounded by three equidistant 3-μL drops of the exponential-phase bacterial suspension. After incubation at 20 °C for 7 − 10 days, photographs were taken. The growth of the pathogenic fungi was assessed by calculating the areas of the mycelium on the digital images using ImageJ. The control (a piece of mycelium with blank LB suspension) and each pair of bacteria and pathogenic fungi were replicated 3–4 times.

Antagonism was expressed as the percentage of mycelial growth inhibition calculated by the formula ([*R*_*mc*_ – *R*_*exp*_] / *R*_*mc*_) × 100%, where *R*_*mc*_ represents the mean mycelial area of the control fungus, and *R*_*exp*_ is the area of the examined pathogenic fungus co-cultured with the examined *Bacillus* (Michereff et al. 1994). For each of the three pathogenic fungi, the significance of antagonism of each of the bacteria was examined by comparing the mycelial area with the respective control using Student's *t* test, and *P* values were adjusted by the Holm–Bonferroni method.

### Adverse effects of bacterial isolates on nematode

To simulate the effect of bacteria on the free-living stage of the plant-pathogenic nematodes, the model nematode, *Caenorhabditis elegans* strain N2 (gift from Dr. Yen-Ping Hsueh, Institute of Molecular Biology, Academia Sinica, Taiwan), was used for the examination. Daily mortality of the nematode was examined in response to hemolytic and non-hemolytic bacterial strains. Synchronized L4 nematodes were cultured on nematode growth medium (NGM; 3 g NaCl, 2.5 g peptone, 17 g agar, 5 mg cholesterol, 1 mL of 1 M CaCl_2_, 1 mL of 1 M MgSO_4_, 25 mL of 1 M KH_2_PO_4_, and H_2_O to 1 L) agar plates seeded with *Escherichia coli* OP50 (gift from Dr. Yen-Ping Hsueh). The examined bacterial isolates were prepared by inoculating in 3 mL of LB liquid broth at 20 °C overnight and then adjusting the OD_600_ value to 0.2. Five treatments were used to examine the survival rate by co-culturing the nematodes with a 20 μL bacterial suspension of 1) the hemolytic bacterial strain, 2) the non-hemolytic bacterial strain, 3) mix of the hemolytic strain + *E. coli* OP50, 4) mix of the non-hemolytic strain + *E*. *coli* OP50, and 5) *E*. *coli* OP50 only (control). For each treatment, 30 L4 larvae were cultured on the NGM agar plate, and the daily survival rate was monitored for 5 days. Each treatment was replicated three times. Survival curves were compared by survival analysis with treatment as the fixed effect and replication as the block. The significance of the fixed effect was assessed by model reduction and the likelihood ratio test. Post hoc multiple comparisons were conducted with Tukey's all-pair comparisons. The model building and hypothesis tests were conducted using the "survival" and "multcomp" packages in R.

## Results

### Bacterial community in infected ant hosts

From the results, a total of 137 bacterial isolates belonging to 133 bacterial strains were obtained from *D*. *thoracicus* infected by *O. pseudolloydii*, with the majority belonging to Bacillota (synonym Firmicutes) and one isolate identified as Actinomycetota (synonym Actinobacteria) (Supplementary file 1). Twenty-seven clades were clustered by the UPGMA method. In total, 10 genera were identified, including *Bacillus*, *Lysinibacillus*, *Neobacillus*, *Oceanobacillus*, *Paenibacillus*, *Paenisporosarcina*, *Peribacillus*, *Priestia*, *Psychrobacillus*, and *Micrococcus*. *Bacillus* was the most abundant and diverse genus, occupying 72.26% of the total bacterial isolates within nine clades. The clade 7 was determined in this study to be *B. cereus* based on the algorithm, which occupied 60.58% of the total bacterial isolates, predominating the bacterial community. In this clade, we found that the 16S rRNA gene sequences based on universal primers have shown a high similarity (> 99%) between *B*. *cereus* and *B*. *thuringiensis*. It is not easy to differentiate *B*. *cereus* from *B*. *thuringiensis* in routine diagnostics, and the identification methods are expensive and laborious. Because the identification was done according to the 16S rRNA gene sequence only in this study, it was labeled here as *B. cereus/thuringiensis* (Fig. S1).

### Hemolytic activity of bacterial isolates

In the 133 bacterial strains*,* 72 (54.14%) displayed the β-hemolysis reaction. None of the isolates displayed an α-hemolysis reaction. The hemolytic strains were detected in eight clades identified as *B. cereus*/*thuringiensis*, *B. massiliogorillae*, *B. subtilis*, *B. thuringiensis*, *Lysinibacillus sphaericus*, *L. xylanilyticus*, *Lysinibacillus* sp., and *Paenibacillus uliginis*. Details of the hemolysis reaction of the bacterial strains are listed in Table S1 and Supplementary file 1.

### Resistance of bacterial isolates to naphthoquinones

**Fig. 2:**
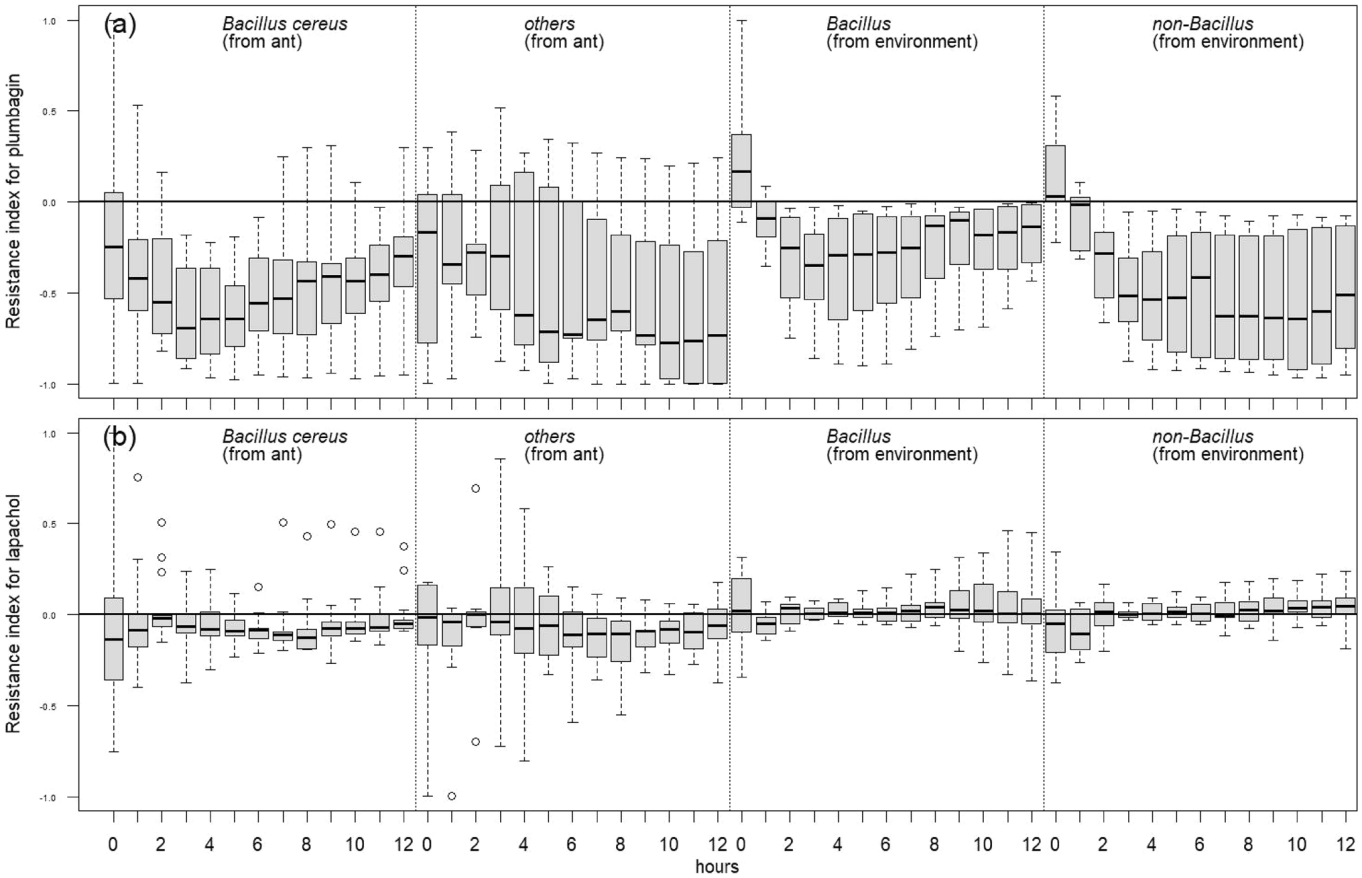
The resistance index of bacterial growth in the presence of two naphthoquinones, lapachol (**a**) and plumbagin (**b**). The results show no significant pairwise differences between *Bacillus cereus*/*thuringiensis* isolated from ant cadavers of *Dolichoderus thoracicus*, seven low-abundant clades (clade 1, 6, 8, 9, 12, 17, 18) isolated from the ant host, *Bacillus* isolates from the environment, and non-*Bacillus* isolates from the environment

Naphthoquinones have previously been known to be produced by *Ophiocordyceps* fungi, and hence, the resistance suggests the advantage of the bacterial isolates to grow in the ant cadaver (Kittakoop et al. 1999). However, for both of the two naphthoquinones, no significant difference was detected among the four groups of the bacteria (Fig. 2). The bacteria from all four groups grew slower in the media containing plumbagin than in the control medium; however, no difference was detected among the four groups (plumbagin: *X*^*2*^ = 4.09, *d.f.* = 3, *P* = 0.2521, Fig. 2a). Conversely, lapachol exerted no obvious influence on the growth of the bacteria compared to the control medium (resistance indexes close to 1). Among the four groups, bacteria from the ant host grew slightly slower relative to the environmental bacteria, but the difference was not statistically significant (lapachol: *X*^*2*^ = 7.16, *d.f.* = 3, *P* = 0.0668, Fig. 2b).

### Hydrolytic enzyme activities by bacterial isolates

All the examined bacterial strains belonging to clade 7 (the predominant species, *B. cereus*/*thuringiensis*) displayed protease activity, whereas some of them displayed lipase activity (8/13), esterase activity (6/13), and chitinase activity (4/13). None of the seven low-abundant clades displayed chitinase activity, two out of the seven displayed protease activity, four displayed lipase activity, and three displayed esterase activity (Table 1).

### Antagonistic activity against pathogenic fungi by bacterial isolates

**Fig. 3:**
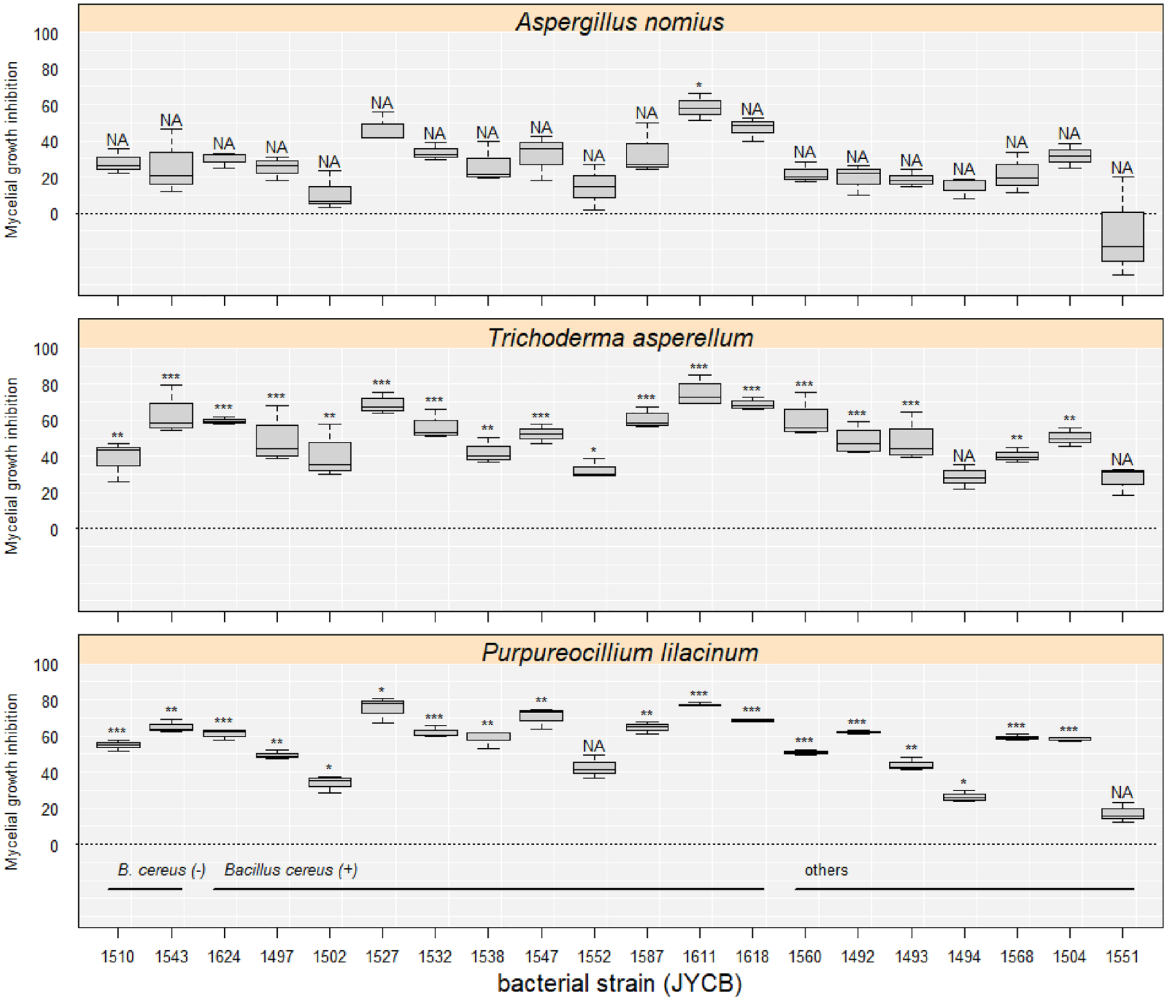
Mycelial growth inhibition (MGI) value of three entomopathogenic fungi under the effects of predominant *Bacillus* strains isolated from ant cadavers infected by *Ophiocordyceps pseudolloydii*. Starts indicate adjusted significant differences compared with "0"

It is not yet possible to culture *O. pseudolloydii* under our laboratory conditions. So far, we are not able to perform the experiment to see whether the bacteria against *O*. *pseudolloydii* itself. Thus, growth inhibition was detected in most of the bacteria strains against the two co-cultured pathogenic fungi (*T. asperellum* and *P. lilacinum*) isolated from the litchi stink bug, whereas there was no influence on the growth of *A. nomius* except JYCB1611 (*Bacillus cereus/thuringiensis*) (Fig. 3). Only two bacteria strains, JCYB1494 (*Oceanobacillus sojae*) and JCYB1551 (*Bacillus* sp.), did significantly inhibit the growth of only one of the pathogenic fungi isolated from the litchi stink bug. One strain, JCYB1552 (*B. cereus/thuringiensis*), did not significantly inhibit the growth of any tested pathogenic fungi. The hemolytic and non-hemolytic strains of the predominant clade, *B. cereus/thuringiensis*, displayed clear growth inhibition against the co-cultured pathogenic fungi from the litchi stink bug.

### Adverse effects of bacterial isolates on nematode

**Fig. 4:**
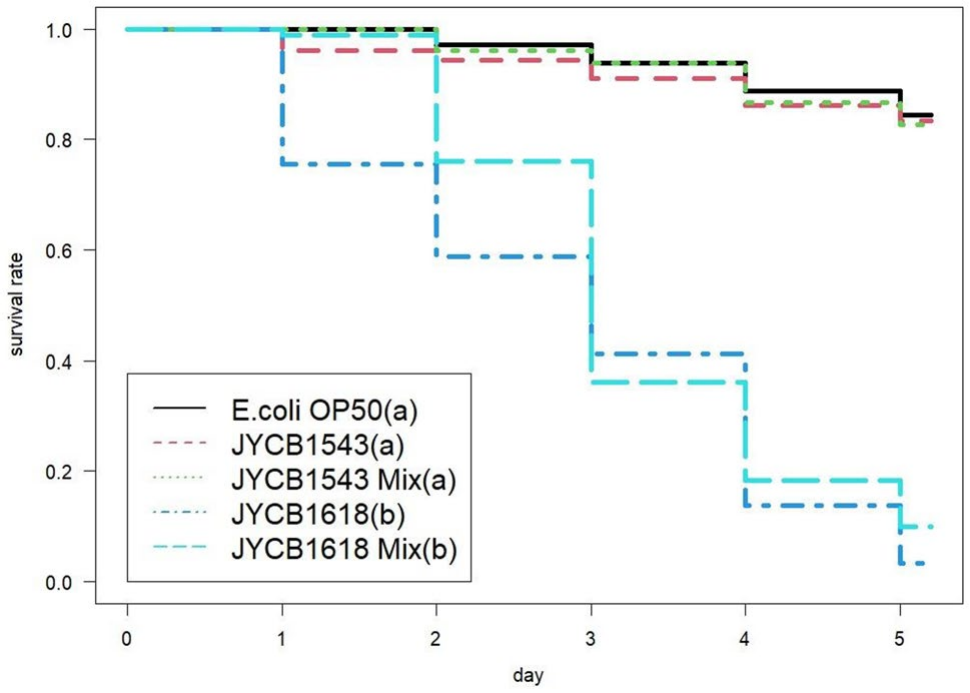
Daily cumulative survival rate of the nematode *Caenorhabditis elegans*, co-cultured with hemolytic (JYCB1618) and non-hemolytic (JYCB1543) bacteria isolated from ant cadavers of *Dolichoderus thoracicus* infected by *Ophiocordyceps pseudolloydii*. Letters indicate significant pairwise differences among the treatments (*p* < 0.05)

The hemolytic *B. cereus/thuringiensis* strain (JYCB1618) displayed a significant adverse effect on the nematode survival (*X*^*2*^ = 636.81, *d.f.* = 10, *P* < 0.001), whereas the non-hemolytic strain (JYCB1543) and the control displayed similar levels of mortality to nematodes (Fig. 4). Mixing of *E. coli* OP50 did not influence the effect of the two *B. cereus/thuringiensis* strains on the nematode survival (Fig. 4).

## Discussion

In this study, we have demonstrated that *B. cereus*/*thuringiensis* predominate the bacterial community isolated from *O*. *pseudolloydii-*infected ant cadavers. Most of the strains of the *B. cereus*/*thuringiensis* isolates are hemolytic, and the activity of hydrolytic enzymes, including chitinase, protease, lipase, and esterase, can be detected in some of the examined strains. Antagonistic activity against a number of environmental/opportunistic fungi with entomopathogenic potential by some isolates of *B. cereus*/*thuringiensis* was also found in the current study, but was less obvious against *A. nomius*. The resistance to naphthoquinone derivatives was not detected in *B. cereus*/*thuringiensis* isolated from the *O. pseudolloydii*-infected ants. In the following paragraphs, we will discuss the role that microbial communities might play in mediating the infection process of *O*. *pseudolloydii* in ants.

### Naphthoquinone resistance

The predominance of *B. cereus*/*thuringiensis* was detected in the ant cadavers colonized with *O. pseudolloydii*. Many species of *Bacillus* exhibit a wide range of physiologic abilities that allow them to live in every natural environment (Cho and Chung 2020; Radhakrishnan et al. 2017). Fungi produce many kinds of bioactive metabolites and have always been considered as a source of new antimicrobial agents (Al-Fakih and Almaqtri 2019). Recent literature has unequivocally shown that fungal secondary metabolites affect the associated microbiome composition and function of the fungi (Deveau et al. 2018; Peleg et al. 2010,). The synthesis and biological evaluation of naphthoquinone derivatives are of great interest since these compounds exhibit strong antibacterial, antifungal, antimalarial, and anticancer activities (López-López et al. 2022). Several naphthoquinone derivatives have been isolated from insects-infected *Ophiocordyceps* fungi (Kittakoop et al. 1999). As a class of bioactive compounds produced by fungi, bacteria, and plants (López López et al. 2014; Medentsev and Akimenko 1998), naphthoquinones are well-known for their antimicrobial activity (de Andrade-Neto et al. 2004; Kittakoop et al. 1999). However, naphthoquinone resistance was not detected in this study. And we do not have any evidence to prove that *O. pseudolloydii* is able to produce naphthoquinone derivatives. Thus, the proliferation of *Bacillus* in ant cadavers cannot be explained by the adaptation of the microbial populations to this antimicrobial compound. However, the naphthoquinones used in this study were those from plants. We cannot exclude the possibility that naphthoquinones from plants do not inhibit fungi. Furthermore, entomopathogenic fungi, including *Ophiocordyceps* spp., also produce polyketides, secondary metabolites with antibiotic activity (Molnar et al. 2010; Wichadakul et al. 2015). These chemicals all have the potential to select sympatric bacteria. In this case, further experiments are needed.

### Production of hydrolytic enzymes

Twenty bacterial strains, including the 13 *B. cereus*/*thuringiensis* strains, were examined for the production of hydrolytic enzymes. Many of them displayed the ability to digest protein and lipid. By contrast, only a few *B. cereus*/*thuringiensis* strains (4/13) were found to hydrolyze chitin. Insect cadavers are ephemeral and nutrient-rich microenvironments. It is not surprising that the bacteria sharing the resources with *O. pseudolloydii* hydrolyze protein and lipid as food resources. However, the impact on the *O. pseudolloydii* infection is currently questionable. The exogenous enzymes could promote the digestion of ant tissue, serving as a public good for the sympatric microbiome. In addition, proteases for host epidermal decomposition might assist *O. pseudolloydii* in killing the host at the end of the infection (Bidochka and Khachatourians 1992; St Leger et al. 1996). Conversely, saprophytic bacteria can also be competitors.

The low number of bacterial strains with the ability to hydrolyze chitin might suggest that the cuticle of the insects is more likely to be the shelter instead of the food resource. Alternatively, the bacteria are might not be important at the step of penetrating insect exoskeleton but rather help the fungus in digesting internal tissues of ants. Another possible role of chitinase is to defend against fungal invaders, given that chitin is a primary component of fungal cell walls (Brzezinska et al. 2014). However, only 1 of the 20 examined bacterial strains displayed a significant antagonistic effect on the three fungi with entomopathogenic potential tested here. This strain (*B. cereus*/*thuringiensis* JYCB1611) is also one of the only four bacterial strains that displayed chitinase activity. Meanwhile, we cannot exclude the hypothesis that the chitinase activity of these associated bacteria is counter-selected to allow them be able to associate with *O*. *pseudolloydii* during the infection process. It might suggest that the microbial community has evolved to have both proteolytic and lipolytic activities to help *O*. *pseudolloydii* to acquire nutrients from the ant cadavers, while it is selected to not produce chitinase in order to avoid the potential antagonistic effect to *O*. *pseudolloydii*.

Bacteria living on or in close proximity to their associated fungal hyphae may positively influence the physiology of the fungal partner. The positive effects can be direct by enhancing fungal growth or improving asexual reproduction or indirect by increasing the nutrients available to its associated fungus (Bastias et al. 2020). If bacteria can provide nutrient support for fungi, then co-occurrence of these associated bacteria would be advantageous to the fungi, which may explain why some bacteria dominate. For example, the arbuscular mycorrhizal fungi can release carbon produced by plant photosynthesis to the environment, thereby promoting phosphate-solubilizing bacterium growth and activity. In return, the phosphate-solubilizing bacterium enhances the mineralization of organic phosphorus, increasing phosphorus availability for the arbuscular mycorrhizal fungi (Zhang et al. 2016). Carbon and phosphorus exchange may enable cooperation between them, and this synergistic interaction can dramatically influence the changes in the bacterial community structure of mycorrhizospheres (Zhang et al. 2018).

The *O*. *pseudolloydii* fungus continually grows inside the ant's body after the ant has died (Chung et al. 2017). Its fruiting body will then sprout from the back of the ant's head and produce spores, which are released onto the forest floor after several weeks. The whole process of fruit body development dramatically increases the cost of fungal growth; therefore, the energy for fungal growth is mostly generated from the infected ants. Thus, the efficiency of the digestive process of macromolecules is crucial to the life cycle of this parasitoid. Conversely, bacterial enzymes (*e*.*g*., chitinase and protease) are also often responsible for the antifungal activity (see next paragraph). In this study, most of the *Bacillus* isolates selected showed hydrolytic enzyme activity and could co-occur synergistically with *O*. *pseudolloydii* by improving the exploitation of the host's resources.

### Antagonism against pathogenic fungi isolated from the insect cadavers

Here, we showed that bacteria isolated from the infected ants displayed obvious antagonism against two of the three examined pathogenic fungi. However, the antagonism was less obvious when co-culturing with *A. nomius*. *Aspergillus nomius* commonly infects social insects, including *D. thoracicus* ants (Lin et al. 2021). Similar to the *Ophiocordyceps* fungi, *A. nomius* also kills the host at the end of the infection and produces conidia on the host cadaver. As observed in ants infected by *Ophiocordyceps* fungi, *Bacillus cereus*/*thuringiensis*, which is likely to invade the ant cadaver from the surrounding environment, may proliferate in the ant infected by *A. nomius*. Thus, it is reasonable that compared to other pathogenic fungi, *B. cereus*/*thuringiensis* had less effect on the growth of *A. nomius*. It is not yet possible to culture *O. pseudolloydii* under our laboratory conditions. However, this result provides indirect evidence that the ant-parasitic fungi might preferentially adapt to environments colonized by *Bacillus* bacteria. Pathogenic fungi isolated from the insect cadavers are potential competitors for the nutrients provided by ant cadavers. The antagonism found in the sympatric bacteria possibly protects the *Ophiocordyceps* fungi from sharing resources with other invaders. This hypothesis is built on the higher tolerance of *O. pseudolloydii* to the sympatric bacteria compared with other possible invaders. Finally, here we found that the hemolytic and non-hemolytic strains of the predominant clade, *B*. *cereus*/*thuringiensis*, displayed clear growth inhibition against the co-cultured pathogenic fungi from the litchi stink bug. This result might suggest that hemolysis is not the crucial trait for competition between the bacteria and these pathogenic fungi.

### Adverse effects of bacterial isolates on nematode

In this study, we found that a hemolytic *B. cereus*/*thuringiensis* strain isolated from the infected ants displayed a significant adverse effect on the nematode (*C. elegans*) survival. Mixing of the regular food, *E. coli* OP50, did not rescue the nematodes from death. Thus, the negative effect is more likely to be caused by the virulence of the bacterium rather than the lack of suitable food. Due to the labor cost, only two strains were examined in this study. It might be an overstatement to suggest that the hemolytic *B. cereus*/*thuringiensis* could play a role in defending the ant cadaver against invasion by saprophytic nematodes. Nevertheless, the antagonism toward sympatric nematodes might be practical in pest control. Plant nematodes are among the important pathogens causing crop losses (Riedel 1988). Most plant nematodes spread and infect new plant hosts during their free-living stage in the soil (Riedel 1988). Nematicides are usually harmful to agricultural environments (Mcsorley 2011). As bacteria are one of the natural enemies of nematodes, spreading bacteria in soil or rhizosphere is one of the efficient ways to control nematodes (Tian et al. 2007). In the current study, the hemolytic *B. cereus/thuringiensis* strain, which is easy to be cultured under laboratory conditions, displayed a significant adverse effect on the nematode (*C. elegans*) survival. More investigation is recommended to determine its potential in the application of nematode control.

## Conclusion

In this study, we found that *B. cereus*/*thuringiensis* predominates the bacterial community of the ant cadaver colonized by *O. pseudolloydii*. Despite no evidence for the source of *B. cereus*/*thuringiensis* in the ant cadaver, most of the strains displayed hemolytic activity, so the bacterium is unlikely to be present in the living ants. *Bacillus* is a main bacterial group in soil (Saxena et al. 2020). Therefore, the saprophytic *B. cereus*/*thuringiensis* could invade the ant cadavers from the surrounding environment. The microenvironments habitated by *O. pseudolloydii* can select for strains of *B. cereus*/*thuringiensis* with compatible or synergistic properties*.* As an invasive bacterium, *B. cereus*/*thuringiensis,* might appear to be a competitor to the *Ophiocordyceps* fungi. However, if the *Ophiocordyceps* fungi are relatively more tolerant to the presence of *B. cereus*/*thuringiensis*, as found in another ant-infecting fungus, *A. nomius*, the possible advantage of *B. cereus*/*thuringiensis* might play an overall role in promoting the fungal infection. Currently, no evidence suggests the mutualism has evolved in the co-evolution between these two organisms, but the mutually beneficial interaction is noteworthy. Finally, it has been shown that the ant-infecting *Ophiocordyceps* genomes are enriched for heat-labile enterotoxins compared to generalist fungal pathogens and have been suggested to play a major role in *Ophiocordyceps* pathogenesis (de Bekker et al. 2017; Kobmoo et al. 2018; Will et al.^2020^). Heat-stable enterotoxins produced by bacteria have also been reported to dysregulate pheromone production in insect fat bodies(Wiygul and Sikorowski 1986; 1991). Finally, further research is required to characterize the microbial communities in the environment surrounding *O*. *pseudolloydii*–ant corpses using second-generation sequencing methods to help us better understand the association between bacteria and this fungus. More phylogenomic and comparative genomic analyses of the fungal and bacterial lineages we reported here should be also conducted in future to better understand the genetic blueprint.

## Supplementary Information

Below is the link to the electronic supplementary material.

Supplementary file1 (DOCX 15 KB)

Supplementary file2 (CSV 12 KB)

Supplementary file3 Fig. S1 Clades of bacterial strains and their estimated taxa from the ant cadavers of Dolichoderus thoracicus. Each of the clades is determined by the sequence dissimilarity (<0.01) according to the UPGMA analysis (TIFF 2591 KB)

## Data Availability

All data generated or analyzed during this study are available from the corresponding author upon request.
