# Supplementary material for: Bacillus predominates in the Ophiocordyceps pseudolloydii-infected ants, and it potentially improves protection and utilization of the host cadavers
Source: Arch Microbiol. 2023 Jan 5;205(1):53. doi: 10.1007/s00203-022-03385-9 (PMC9816197; doi:10.1007/s00203-022-03385-9)
Supplement: Supplementary file 1 — Supplementary file1 (DOCX 15 KB) [file 203_2022_3385_MOESM1_ESM.docx]

**Table S1.** Bacterial clades. **Species** are determined by the BLAST identity with the NCBI database. **Clade** (referring to the codes in Supplementary Fig. S1) is determined by the sequence dissimilarity (<0.01) of the bacterial strains according to the UPGMA analysis. **Abundance** denotes the sum of the colonies belonging to the same clade. **Hemolytic** is marked by +, -, and +/-, representing the hemolytic activity displayed in all, none, or some of the bacterial strains belonging to the same clade.

| **Genus** | **Species** | **Clade** | **Abundance** | **Hemolytic** |
| --- | --- | --- | --- | --- |
| **Phylum Firmicutes** |  |  |  |  |
| *Bacillus* | *B. cereus/thuringiensis* | 7 | 83 | -/+ |
|  | *B. circulans* | 10 | 1 | - |
|  | *B. gibsonii* | 6 | 1 | - |
|  | *B. licheniformis* | 24 | 1 | - |
|  | *B. massiliogorillae* | 20 | 1 | + |
|  | *B. subtilis* | 17 | 9 | -/+ |
|  | *B. thuringiensis* | 25 | 1 | + |
|  | *Bacillus* sp. | 14 | 1 | - |
|  | *Bacillus* sp. | 18 | 1 | - |
| *Lysinibacillus* | *L. contaminans* | 11 | 4 | - |
|  | *L. sphaericus* | 26 | 3 | -/+ |
|  | *L. xylanilyticus* | 12 | 6 | -/+ |
|  | *Lysinibacillus* sp. | 13 | 1 | + |
| *Neobacillus* | *N. novalis* | 22 | 3 | - |
| *Oceanobacillus* | *O. sojae* | 9 | 1 | - |
| *Paenibacillus* | *P. agarexedens* | 5 | 1 | - |
|  | *P. amylolyticus* | 2 | 1 | - |
|  | *P. taichungensis* | 1 | 4 | - |
|  | *P. uliginis* | 4 | 2 | -/+ |
|  | *Paenibacillus* sp. | 3 | 1 | - |
|  | *Paenibacillus* sp. | 27 | 1 | - |
| *Paenisporosarcina* | *Paenisporosarcina* sp. | 15 | 2 | - |
| *Peribacillus* | *P. butanolivorans* | 23 | 3 | - |
|  | *P. muralis* | 19 | 1 | - |
| *Priestia* | *P. megaterium* | 8 | 2 | - |
| *Psychrobacillus* | *Psychrobacillus* sp. | 16 | 1 | - |
| **Phylum Actinobacteria** |  |  |  |  |
| *Micrococcus* | *M. luteus* | 21 | 1 | - |
