# Supplementary figures and images for: Bacillus predominates in the Ophiocordyceps pseudolloydii-infected ants, and it potentially improves protection and utilization of the host cadavers
Source: Arch Microbiol. 2023 Jan 5;205(1):53. doi: 10.1007/s00203-022-03385-9 (PMC9816197; doi:10.1007/s00203-022-03385-9)

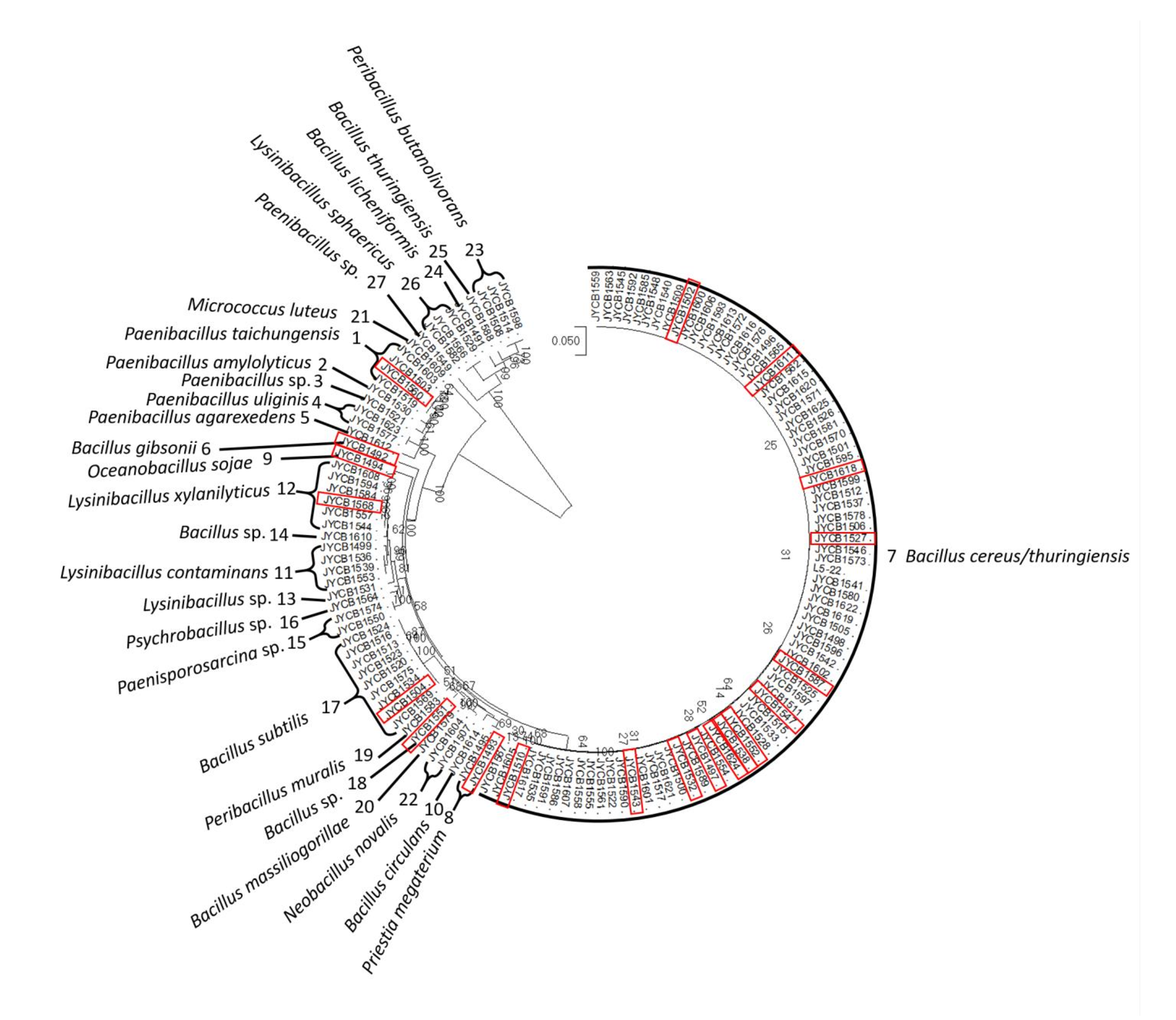

Supplement: Supplementary file 3 — Supplementary file3 Fig. S1 Clades of bacterial strains and their estimated taxa from the ant cadavers of Dolichoderus thoracicus. Each of the clades is determined by the sequence dissimilarity (<0.01) according to the UPGMA analysis (TIFF 2591 KB) [file 203_2022_3385_MOESM3_ESM.tiff]
